# Mobile phones support adherence and retention of indigenous participants in a randomised controlled trial: strategies and lessons learnt

**DOI:** 10.1186/1471-2458-14-622

**Published:** 2014-06-18

**Authors:** Gabrielle B McCallum, Lesley A Versteegh, Peter S Morris, Clare C Mckay, Nerida J Jacobsen, Andrew V White, Heather A D’Antoine, Anne B Chang

**Affiliations:** 1 Child Health Division, Menzies School of Health Research, Charles Darwin University, Darwin, Northern Territory, Australia; 2 Department of Paediatrics, Royal Darwin Hospital, Darwin, Northern Territory, Australia; 3 Department of Paediatrics, The Townsville Hospital, Townsville, Queensland, Australia; 4 Queensland Children’s Respiratory Centre, Queensland Children’s Medical Research Institute, Royal Children’s Hospital, Brisbane, Queensland, Australia

**Keywords:** Mobile phones, SMS, Adherence, Randomised controlled trial, ALRTI, Bronchiolitis, Indigenous

## Abstract

**Background:**

Ensuring adherence to treatment and retention is important in clinical trials, particularly in remote areas and minority groups. We describe a novel approach to improve adherence, retention and clinical review rates of Indigenous children.

**Methods:**

This descriptive study was nested within a placebo-controlled, randomised trial (RCT) on weekly azithromycin (or placebo) for 3-weeks. Indigenous children aged ≤24-months hospitalised with acute bronchiolitis were recruited from two tertiary hospitals in northern Australia (Darwin and Townsville). Using mobile phones embedded within a culturally-sensitive approach and framework, we report our strategies used and results obtained. Our main outcome measure was rates of adherence to medications, retention in the RCT and self-presentation (with child) to clinic for a clinical review on day-21.

**Results:**

Of 301 eligible children, 76 (21%) families declined participation and 39 (13%) did not have access to a mobile phone. 186 Indigenous children were randomised and received dose one under supervision in hospital. Subsequently, 182 (99%) children received dose two (day-7), 169 (93%) dose three (day-14) and 180 (97%) attended their clinical review (day-21). A median of 2 calls (IQR 1–3) were needed to verify adherence. Importantly, over 97% of children remained in the RCT until their clinical endpoint at day-21.

**Conclusions:**

In our setting, the use of mobile phones within an Indigenous-appropriate framework has been an effective strategy to support a clinical trial involving Australian Indigenous children in urban and remote Australia. Further research is required to explore other applications of this approach, including the impact on clinical outcomes.

**Trial registration:**

ACTRN12608000150347 (RCT component).

## Background

In the Northern Territory (NT), Indigenous children have high hospitalisation rates of bronchiolitis (352 per 1000) and more severe disease. Most children admitted are retrieved from remote communities [1,2]. Hospitalised episodes of lower respiratory infections are associated with later development of chronic lung disease [3,4]. In an attempt to improve clinical outcomes, we conducted a double blind randomised controlled trial (RCT) (using azithromycin) [5] within an evidence-based framework for assessing and prioritising health interventions. RCTs are accepted as the highest level of evidence available. However, the lack of appropriate RCTs may contribute to poor participation, attrition and treatment inequalities in minority groups [6]. While some progress has been made in reducing health disparities, there is a continued need for intervention studies, both prevention and treatment trials, that focus on minority population(s) [7].

There are several possible methods that can be used to increase the adherence and reduce attrition (increase retention) in RCTs. One such method is the use of mobile phones as a means of communication. Mobile phones offer the advantage of real time communication, do not require high skills to function, are easily accessible, affordable and not restricted to computer or land line access [8]. The number of published research using the short message service (SMS) component of mobile phones to evaluate a range of health conditions has increased. However, the conditions studied have commonly focused on adult disease surveillance and chronic diseases [9-12]. Data on SMS outcomes in paediatric conditions; [13] i.e. acute illnesses, Indigenous populations or remote areas are limited.

In this study, we report on a novel approach to improve adherence, retention and clinical follow-up post-hospitalisation in 186 Australian Indigenous children participating in a RCT.

## Methods

### Study design

This study is embedded within a double-blinded, placebo-controlled, RCT conducted at the Royal Darwin Hospital and The Townsville Hospital between June 2010 and September 2013. We briefly describe the RCT below as the protocol has been published [5]. The RCT examines the question: ‘amongst children hospitalised with acute bronchiolitis, does azithromycin (compared to placebo) given once/week for three doses improve clinical outcomes?’ For this study, we describe the cohort of children enrolled in this RCT, strategies used and results obtained in ensuring adherence, retention and presentation to the clinic for follow-up. The trial was approved by each institution’s Human Research Ethics Committee and was registered with the Australian and New Zealand Clinical Trials Register: Clinical trials number: ACTRN12608000150347.

### Study population

Children were eligible if they were Indigenous, aged ≤24months, admitted to hospital with a clinical diagnosis of acute bronchiolitis, recruited within 24 hours of admission. There was also a requirement for the parent to have a mobile phone.

### Recruitment and retention approach

Research nurses visited the paediatric wards twice daily to screen recently admitted children. Only parents whose child met eligibility criteria were approached. A summary of our frame work is presented in Table 1. Often parents had come to hospital in the early hours of the morning, were sleep deprived and had not retained information hospital staff provided. Therefore, research nurses always provided additional education on bronchiolitis using a pictorial-based flipchart (http://www.menzies.edu.au/page/Resources/Bronchiolitis_Lower_respiratory_tract_infection/). Time was spent with parents discussing the treatment and management of bronchiolitis and what to expect post discharge, regardless of the decision to be involved in the RCT. This appeared to enhance relationships and trust. Only when parents understood what bronchiolitis was, did research nurses proceed with discussion about the RCT. A pictorial consent flipchart was used in conjunction with a plain language information booklet (endorsed by the Menzies Child Health Indigenous Reference Group), to assist in the consent process. The time from screening to enrolment was recorded.

**Table 1:** Framework used in our study

**Pre study discussion**	● Indigenous Reference Group (IRG) (consultation and endorsement of study)
	● Data Safety Monitoring Board (DSMB) (endorsement of study plan)
**On the ground**	● Clinical Nurses with broad experience working in
	● Indigenous health
**Research team**	● Paediatrics
	● Clinical research
	● Remote health settings
**Project specific**	● Briefings to IRG on study progress.
	● DSMB updates on recruitment and retention.
	● Providing education on bronchiolitis to parents using pictorial flipchart.
	● Research nurses spending time discussing child’s treatment and management in hospital and home.
	● Consent process: using a pictorial flipchart in conjunction with a plain language information booklet.
	● Education on how to prepare, when to give medication and attend health clinic for 21 day review.
	● Education to nursing staff on paediatric wards to improve awareness and understanding of bronchiolitis.
**Mobile phone specific**	● Calling parent in hospital (number transcribed correctly and enabled two way communication).
	● Obtaining additional number (if able).
	● Calling parent from personal/study mobiles.
	● Providing parents with option of calling from free 1800 number.

Once written informed consent was obtained from the parent or guardian, children were randomised to receive either azithromycin or placebo. The first dose was directly supervised in hospital; the remaining two doses were supervised by research nurses (urban-based children) or given at home by parents (remote-based children) (between days 5–9 and 10–12). The endpoint was a clinical review on day-21 (between days 20–30) by research nurses (urban-based children) or at the local health clinic (remote-based children) to determine presence of persistent respiratory symptoms and signs. Remoteness was defined as more than 100 km from a tertiary hospital.

Standardised assessment forms were used to collect clinical information from each child. Prior to discharge, parents were shown how to constitute the medication and were given the remaining medications in a sealed plastic bag which included syringes, 10 ml sterile water vials and a fridge magnet (with reminders when each medication and the clinical review was due).

We advised parents that we would ring or SMS when children were due to receive the medications and attend the clinic for their clinical review (remote-based children) or visit at home (urban-based children). For remote-based children, a phone call was also made to the local health clinic explaining the child’s involvement in the RCT and follow up required as part of routine clinical care post hospitalisation. A template was faxed to the health clinic and faxed back after the clinical review was completed. The number of contacts and reasons why contact could not be made were recorded (if applicable). A $20 mobile recharge voucher was sent via SMS after the third dose (but before clinical review) to thank parents for their participation.

### Other strategies used

A number of strategies were implemented to help maintain contact with parents throughout the RCT. Firstly, research nurses called parent’s mobile phones prior to discharge. This ensured the number was transcribed correctly and started mobile phone contact while still meeting in person. Secondly, we obtained an additional mobile number for occasions when we were unable to contact the parent. Thirdly, we identified that parents would rarely answer phone calls from a blocked (unknown) number. Research nurses therefore called from their personal mobiles (or a study mobile). Parents also had the option to call research nurses on the free 1800 number if they had any questions or concerns. However, we did not receive any call on this number. Parents preferred to call the personal mobiles of the research nurses.

### Statistical analysis

Data were entered on an Access database and analysed using Stata version 12 (Stata corp College Station, Texas, USA). Data are presented as numbers and percentages, median and interquartile range (IQR 25-75% and or range). We describe feedback from parents and staff experiences in text.

## Results

### Demographics

**Figure 1:**
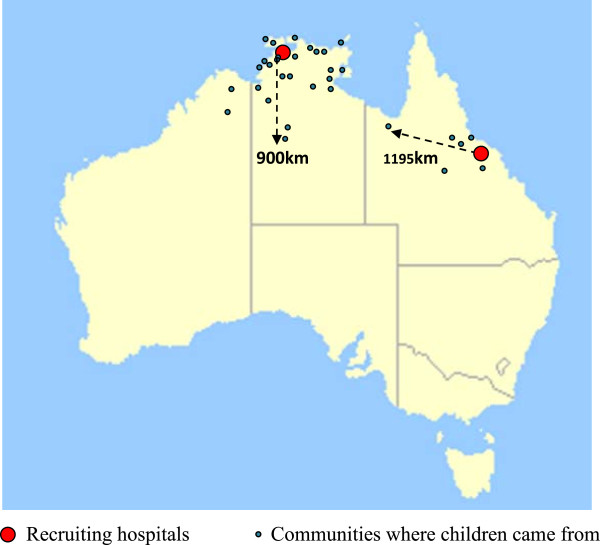
**Map of communities.** NB: Some communities appear to be located in the ocean, however are Islands north of the mainland.

Of 301 eligible children, 76 (21%) families declined participation and 39 (13%) did not have access to a mobile phone. A total of 186 children were enrolled; 161 in Darwin and 25 in Townsville. The median time taken to enrol participants was 30 minutes (range 20 minutes – 5 hours). The median age was 5.4 months (IQR 3–9); 111 (60%) boys, and 75 (40%) girls. Four children were withdrawn from receiving further medications (n = 3 for dose 2 and n = 4 for dose 3) by the paediatric team at site hospitals due to other medical reasons. The remaining children were followed up until they reached their endpoint (day-21 clinical review). More than two thirds of the children 144 (70%) lived in remote Indigenous communities. Of the Darwin-based cohort, 139 (85%) children were from remote-based communities. In contrast, only 5 (20%) children enrolled in Townsville were remote-based. Figure 1 illustrates approximate locations of all communities and distances from site hospitals.

### Medication and clinical review

All children 186 (100%) received the first dose of medication in hospital. A small number of children received dose-2 (n = 17 (8%)) and dose-3 (n = 3 (1%)) in hospital. For the remainder, research nurses made contact with parents on their mobiles when medication(s) and the clinic review were due. The adherence, retention and follow-up rate for the entire cohort was very high. Overall, 182 (99%) children received dose two (day-7), 169 (93%) received dose three (day-14) and 180 (97%) children attended their day-21 clinical review. Table 2 summarises the number of medication doses received, clinic reviews attended, missed and the median number of phone calls required to contact the carer.For dose-2, 62% of parents were able to be contacted on the first attempt; this reduced to 39% by dose-3 and 18% for the clinical review (Figure 2). However, only a small number of calls were needed to verify when medication(s) and the clinical review were completed (median 2 calls (IQR 1–3)). Reasons for calls not being taken were most frequently due to (i) mobile phones being turned off; (ii) mobile phones not charged; or (iii) parents not answering a call from a blocked (private) number.

**Table 2:** Medication doses and clinic review by site

**Trial procedures**	**Darwin**			**Townsville**		
	**Given (n = 161)**	**Missed N (%)**	**Number contacts median (range)#**	**Given (n = 25)**	**Missed N (%)**	**Number contacts median (range)**
**Dose 1**	161 (100%)	0 (0%)	N/A	25 (100%)	0 (0%)	N/A
Dose 2*	157 (98%)	4 (2%)	1 (1–12)	25 (100%)	0 (0%)	2 (1–5)
Dose 3*	147 (91%)	14 (9%)	2 (1–13)	22 (88%)	3 (12%)	2 (1–6)
**Clinical review**	156 (97%)	5 (3%)	3 (1–17)	24 (96%)	1 (4%)	3 (1–7)

*3 children withdrawn from dose 2, 4 children withdrawn from dose 3 by the medical team.

#Combination of phone calls/SMS.

**Figure 2:**
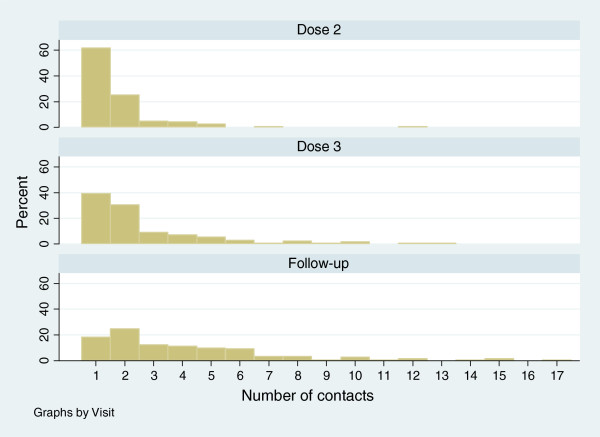
Number of contacts medication dose and clinical follow up.

## Discussion

In our setting, it appears that mobile phones, combined with a culturally sensitive approach, were a simple and effective tool to facilitate adherence in a clinical trial. To our knowledge, this is the first RCT involving Indigenous children that has used mobile phones to support adherence to research protocols. The success of our strategies is documented by a 97% retention rate, the highest we have ever achieved in a setting that involved children in the community.

The use of mobile phones in studies is not new. Previous research has shown mobile phones can have important benefits for clinic attendance, adherence to medications and treatment plans [14-17]. However we found only 3 studies involving children and none were relevant to Indigenous Australians or in acute illnesses [13,18,19] Two of the 3 studies related to immunisations, [13,18] and the third was on reminders for appointments before and after cataract surgery in a large Chinese city hospital [19]. Two studies reported an improvement in the intervention group, compared to controls 43% vs. 39.9% [18] and 91% vs. 62% respectively [19]. The third study reported similar adherence in both groups using an intention to treat analysis 66% vs. 68% [13]. In contrast to the above studies, our study is not a RCT on mobile phones but a unique report on how we achieved an exceptional high retention and follow-up rate in a study setting where adherence to medications and follow-up has been reported to be generally difficult. While we were unable to observe adherence with doses 2 and 3 for remote-based children (we were reliant on parents providing this information), the day-21 follow-up rate of >97% at the local health clinic provides evidence of the success of our approach.

Including minorities in RCTs is important in addressing health gaps [20]. Adherence has been reported to be particularly challenging in those who are socially disadvantaged communities [7]. Improving adherence and reducing attrition is important in all clinical trials. Strategies to reduce attrition have the potential to increase power and generalisability of results [21]. Our study has also shown that adherence to medications in the community setting is feasible, thus the opportunity for community based clinical care and follow-up can be highly successful. In addition to our mobile phone strategies, appropriate measures include: (i) building relationships and trust with parents; (ii) using culturally appropriate educational material; and (iii) personal contact with parents. It may also be important that all research staff were paediatric-trained with experience in working with Indigenous parents and children.

Our mobile phone strategy not only included obtaining multiple phone numbers but also calling from a mobile that displayed a number that could be identified by the parent. Over the past 14 years, network coverage in remote Australia has substantially improved. A study in the NT reported that mobile phones have become an essential part of relaying information to family members who were travelling or away from home [22].

Our strategies and findings have to be interpreted in the context of our target population and study settings. We recruited only children whose parents had a mobile phone as geographical remoteness limited our options to ascertain adherence. Although we did not expect the high number of mobile phone ownership, we found that only a small number of parents (13%) did not have access to a mobile phone at time of recruitment. It was not feasible for us to request community health clinics to supervise medication dosing as most of the children come from remote clinics with very high workloads. The clinical review was attended by health clinic staff as part of best practice guidelines for routine clinical care post hospitalisation for a respiratory infection in Australia and many affluent countries.

Families received a $20 mobile recharge voucher after the final medication dose, to thank them for their participation. While we provided this incentive, we do not feel this was fundamental to the adherence and retention of participants in our trial. Importantly, the incentive was provided before the day-21 clinical review, where the presentation rate was 97%. Previously, incentives in clinical trials have only reported small improvements in participant retention between 2-13% [21,23]. One RCT involved SMS reminders and provided a $20 gift card at time of enrolment [24]. The RCT [24] described that gift cards were not important to 22% of participants, somewhat important in 50%, and very important to 28% with regard to their participation in the RCT [13].

We speculate that building relationships and trust were fundamental to our high success of adherence and retention in this trial. In general, parents expressed how they felt supported in hospital and at home, knowing that our staff were there to talk to if they had queries or concerns about their child. In our setting, displacement to a major teaching hospital from a remote community can be distressing for Indigenous people. The approach used by our research nurses helped alleviate parent’s anxiety by providing support and understanding of bronchiolitis and thus we feel fundamental to them continuing in the trial until the child’s endpoint. This was part of our culture-appropriate framework (Table 1). Our framework is supported by a similar strategies used to enhance participation of Maori people in a cardiovascular-based RCT in New Zealand. The NZ study outlined the importance of involving experienced Maori researchers at each time point of the trial, employing experienced Maori researchers, who used culturally specific processes for participation and retention of Maori participants and ongoing contact with Maori researchers and participants [24]. Such frameworks are important and highlight the effectiveness of strategies that are culturally appropriate, thus improving the participation and retention rates in minority populations.

Within our framework, we implemented multiple strategies to support adherence and retention of participants. It is difficult to ascertain the relative contribution to these strategies. This study was embedded within an RCT, thus is complex with the respect to the possible interaction between both a treatment intervention (azithromycin or placebo) and enhancing support (implementing cultural framework). Future treatment trials should account for these factors. One of our study’s limitations includes the lack of in-depth qualitative data to explore this issue. Also, our intervention period is relatively short (3 weeks). Whether or not these strategies will also be successful in longer term interventions remains unknown. Although the data presented are not high-level evidence (i.e. not a RCT), we have shown that the use of strategies employed here has led to an exceptionally high adherence and retention rate. This may have implications for clinical service in remote Indigenous settings and may improve health outcomes. It should be further studied as provision of high quality clinical service and ensuring adherence is a challenge in many settings, particularly in remote Indigenous settings.

## Conclusions

Our data have provided important and novel data that the use of mobile phones, in conjunction with a culturally sensitive approach, is an effective strategy to support clinical trial protocols in Indigenous children living in urban and remote Australia. There is an opportunity to use these strategies to support health service delivery in remote communities that may improve adherence to medications and clinic attendance. Further research is required to explore the feasibility in these setting for health outcomes, cost effectiveness and long term sustainability using our described framework.

## Abbreviations

ACTRN: Australian New Zealand Clinical Trials Registry; IQR: Interquartile range; NT: Northern territory; RCT: Randomised controlled trial; SMS: Short message service.

## Competing interests

The authors declare that they have no financial competing interests.

## Authors’ contributions

GBM set up and coordinated the study, recruited participants, performed the data analysis and drafted the manuscript. LAV, CCM, NJ, AVW recruited participants and edited the manuscript. ABC, PSM conceptualised the study, interpreted the data and edited the manuscript. HD provided cultural integrity support. All authors contributed to the study design and critically reviewed the manuscript and approved the final version.

## Pre-publication history

The pre-publication history for this paper can be accessed here:

http://www.biomedcentral.com/1471-2458/14/622/prepub
